# Preparation and stability of silver/kerosene nanofluids

**DOI:** 10.1186/1556-276X-7-362

**Published:** 2012-07-02

**Authors:** Dan Li, Wenjun Fang

**Affiliations:** 1 Department of Chemistry and Chemical Engineering, Weifang University, Weifang, Shandong Province, 261061, China; 2 Department of Chemistry, Zhejiang University, Hangzhou, Zhejiang Province, 310027, China

**Keywords:** Silver nanofluids, Dialkyl dithiophosphate, Kerosene, Thermal oxidation

## Abstract

A series of silver nanoparticles surface-coated with di-*n*-dodecyldithiophosphate, di-*n*-cetyldithiophosphate, or di-*n*-octadecyldithiophosphate have been prepared and have good dispersity in alkanes or kerosene. Stable silver nanofluids can be formed in alkanes or kerosene with the surface-coated silver nanoparticles. Thermal stability of the silver nanofluids has been measured at different temperatures. The effects of the silver nanoparticles on the thermal oxidation of kerosene have been investigated at different temperatures. The coatings can be released from the surface of the silver nanoparticles above 150°C, giving oxygen access to the silver core and inhibiting the kerosene oxidized by oxygen.

## Background

Nanofluids, nanometal, or metal oxide particles suspended in traditional fluids (water, ethylene glycol, and engine oils) are receiving much more attention in recent years because of the advantages of their heat transfer capacities and broad application prospects [1-5]. Metal nanoparticles with low dispersity in paraffin oils or nonpolar organic solvents show their limited applications. Nanofluid production faces major challenges, such as poor dispersity of metal nanoparticles, agglomeration of particles in base fluids, and the rapid settling of particles in fluids [6].

Silver nanoparticles have been applied in many science and technology areas such as antimicrobial materials, photonics, surface-enhanced Raman scattering, electronics, and catalysis [6-11]. Sometimes, silver nanoparticles with low dispersity in oils or nonpolar organic solvents show their limited applications. Surfactants and extractants such as oleic acid [7,8], alkylthiol [9,10], alkylamine [11], and di(2-ethylhexyl) phosphoric acid [12] have been employed to coat or modify the surfaces of the metal nanoparticles to improve the dispersity in paraffin or oils. Dialkyl dithiophosphates (DDP, (RO)_2_PS_2_^−^), which are composed of nonpolar alkyl hydrocarbon tails and polar head groups, are appropriate for coordinating with the metal nanoparticle surfaces. Dialkyl dithiophosphates and their derivatives are versatile ligands. Metal complexes with DDP have been widely used in application as antioxidants, lubricant additives, and bioactive agents [13-15].

This work describes the preparation of a series of surface-coated silver nanoparticles using dialkyl dithiophosphates with different hydrocarbon chain lengths (C_12_, C_16_, C_18_) as efficient bonding ligands. Differences of the nanoparticle size, size distribution, and hydrophobicity have been investigated. The thermal stability of silver-kerosene nanofluids is studied. The effects of the silver nanoparticles on the thermal oxidation stability of kerosene are discussed by monitoring the hydroperoxide formation during the thermal oxidation. The results may provide important information on the study and application of additives of oil and fuel.

## Methods

### Materials

Silver nitrate, ascorbic acid, cyclohexylamine, and ethanol which are analytically pure reagents were purchased from Sinopharm Chemical Reagent Co., Ltd., Shanghai, China. The coating ligands, pyridinium di-*n*-dodecyldithiophosphate (DDP12), pyridinium di-*n*-cetyldithiophosphate (DDP16), and pyridinium di-*n*-octadecyldithiophosphate (DDP18) were synthesized in our laboratory according to the literature procedures [14].

### Preparation of nanoparticles

The following typical preparation process is described. A mixture is formed with 0.5 mmol dialkyl dithiophosphate dissolved in a mixed solvent of deionized water, ethanol, and cyclohexylamine. The silver nitrate solution (2 mmol, 20 mL) was added into the above mixture, which was heated up to 60°C under stirring. Excess ascorbic acid (0.05 g/mL, 20 mL) was then added, and the reaction was allowed to last for 3 h under vigorous stirring. After that, the mixture was moved to be cooled and kept at the ambient temperature. The powders were separated by centrifugation and washed with ethanol and water several times. Finally, the surface-coated nanoparticles were dried in a vacuum oven at 40°C. A series of nanoparticles were synthesized by similar preparation methods with different ligands and reaction temperatures. The nanoparticles are dispersed in nonpolar solvents such as chloroform, dichloromethane, or kerosene to form different nanofluids or colloids.

### Characterization

The X-ray diffraction (XRD) patterns of silver nanoparticles were recorded by employing a Bruker D8 Advance X-ray diffractometer (Bruker Optik GmbH, Ettlingen, Germany) with monochromatized Cu Kα radiation (*λ* = 1.5405 Å). A transmission electron microscope (TEM; CM-200, FEI-Philips, Hillsboro, OR, USA) was used to determine the size and morphology of the silver nanoparticles. Samples were dispersed in dichloromethane and prepared by placing a drop on a carbon-coated standard copper grid. The particle size distribution was analyzed by measuring over 200 particles from the TEM micrographs and using a digital micrograph software. The silver substrates were prepared by depositing several drops of silver nanoparticle suspension over glass slides and waiting for solvent evaporation. The metal content of the silver nanoparticles was determined by thermogravimetry (TG) analysis. Measurements of the particle absorption band were performed using a UV-1770 spectrophotometer (Shimadzu Corporation, Nakagyo-ku, Kyoto, Japan) equipped with 1.0-cm quartz cells at 25°C.

### Thermal oxidation

The silver-kerosene nanofluids (0.1 mass% silver nanoparticles) were thermally oxidized in an isothermal apparatus [16]. Each test tube containing a 100-mL sample of silver nanofluids was placed in the heated test well. The flow meter was used to regulate the oxygen flow with the rate of 30 mL/min into each sample through a gas dispersion tube. The investigated samples were subjected to thermal oxidation at 120°C, 140°C, and 150°C. A small aliquot (<0.5 mL) of the samples was removed from the test tubes at fixed time intervals for the hydroperoxide measurements. The hydroperoxides formed in the samples during the thermal oxidization process were determined by measuring the absorption spectra of the iodine-starch solutions [16,17] using ultraviolet–visible (UV–vis) spectrometry.

## Results and discussion

### XRD, TEM, TG, and UV–vis analyses of silver nanoparticles

**Figure 1:**
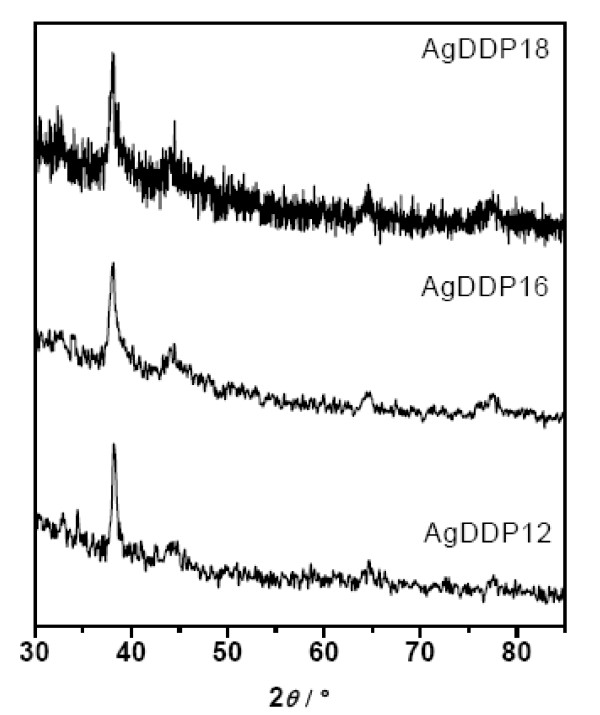
XRD patterns of surface-coated silver nanoparticles with dialkyl dithiophosphates.

XRD was employed to examine the crystalline structures of the silver nanoparticles coated with DDP of various hydrocarbon chain lengths (C_12_, C_16_, C_18_), which were prepared at 60°C. For all the samples, the XRD peaks in Figure 1 can be well assigned to the (111), (200), and (220) crystallographic planes of face-centered cubic (fcc) silver crystals, respectively.

**Figure 2:**
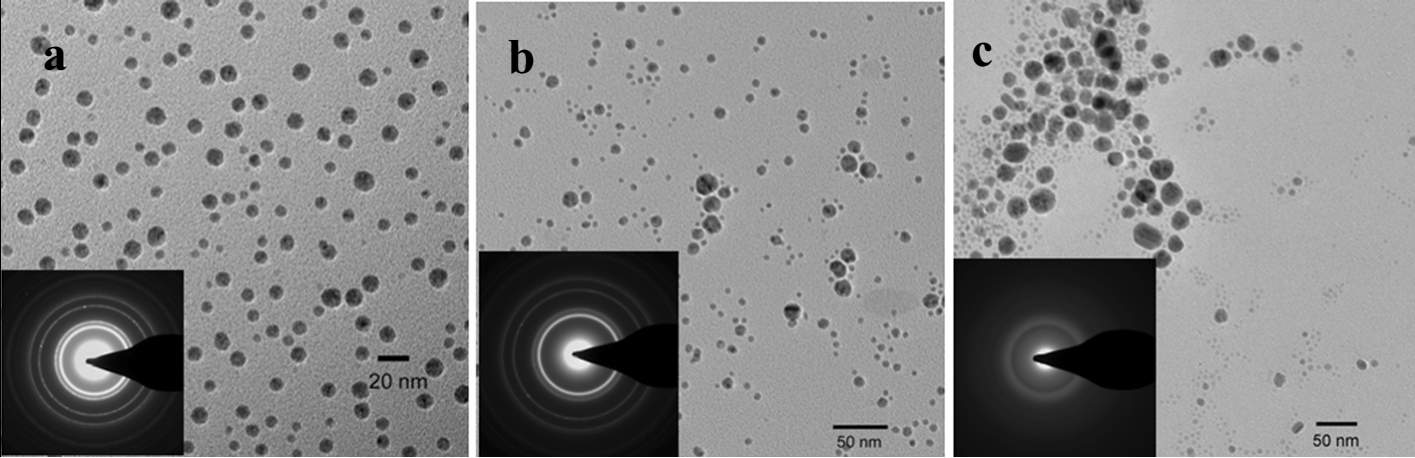
**TEM and SAED images of surface-coated silver nanoparticles at 60 °C.** (**a**) AgDDP12, (**b**) AgDDP16, and (**c**) AgDDP18.

The prepared silver nanoparticles were dispersed in CH_2_Cl_2_ for the characterization of the particle size and size distribution through TEM observations. Figure 2 shows typical TEM images and selected area electron diffraction (SAED) pattern of the silver nanoparticles coated with DDP at 60°C. The SAED pattern of the sample shows fcc ring patterns. The TEM image of AgDDP12 (Figure 2a) shows spherical growth of silver nanoparticles and displays a size distribution ranging from 8 to 12 nm. The nanoparticles are well separated from each other, and the aggregation of the silver core is effectively suppressed by the ligands. The size distribution of AgDDP16 (Figure 2b) is from 3 to 20 nm, which is relatively broader than that of AgDDP12, while the particles still remain well-separated. The TEM image of AgDDP18 (Figure 2c) shows that its size distribution (ranging from 3 to 31 nm) is larger than that of AgDDP12 or AgDDP16. These particles are polydispersed, and the morphologies are not regularly spherical, but distorted. The results indicate that the surface coating ligands with different chain lengths impact the formation and size of silver nanoparticles. The mixed solvent of deionized water, ethanol, and cyclohexylamine is used for stabilizing Ag^+^ and ligands in the reaction system of the coexisting aqueous phase during the preparation of silver nanoparticles. The silver nanoparticles were reduced by ascorbic acid in the reaction system. With the silver cores forming, the ligands (dithiophosphate) coated the surface of the silver cores quickly and separated from the reaction system. Dithiophosphates act as a surfactant to coat the newly generated particle surface, coordinating their P = S groups on the newly generated silver particle surface; the hydrophobic carbon tails of the dithiophosphates are pointed outwards from the surface of the particles. Dithiophosphates are used to control the growth of silver particles and to prevent their agglomeration. The size and dispersibility of the obtained silver nanoparticles can be affected by the chain length of dithiophosphates.

The silver nanoparticles, AgDDP16 and AgDDP18, were synthesized by a similar procedure for AgDDP12. The growth of the metallic silver core was suppressed by the addition of dithiophosphates. In the preparation process, dithiophosphates coated quickly on the surface of the silver core, and the silver nanoparticles with coating layer have different solubilities in the reaction system, which is a mixture of water, ethanol, and cyclohexylamine. The solubility of the three dialkyl dithiophosphates of various chain lengths (C12, C16, C18) in the reaction system was different at 60°C. The dithiophosphate ligands with longer chain length (DDP16 and DDP18) are not completely soluble in the mixed solution of cyclohexylamine/water at 60°C. It had difficulty in capping the surface of the newly generated particles, which may make the silver nanoparticles to be partly coated, and the particle size of AgDDP16 and AgDDP18 was inhomogenous. So, the size of the silver particles was not controlled well by DDP16 and DDP18. At 60°C, AgDDP18 separated more quickly than AgDDP16 and AgDDP12. Therefore, the size distributions of the silver nanoparticles coated with longer chain ligands were wider than those coated with shorter chain ligands at 60°C.

**Figure 3:**
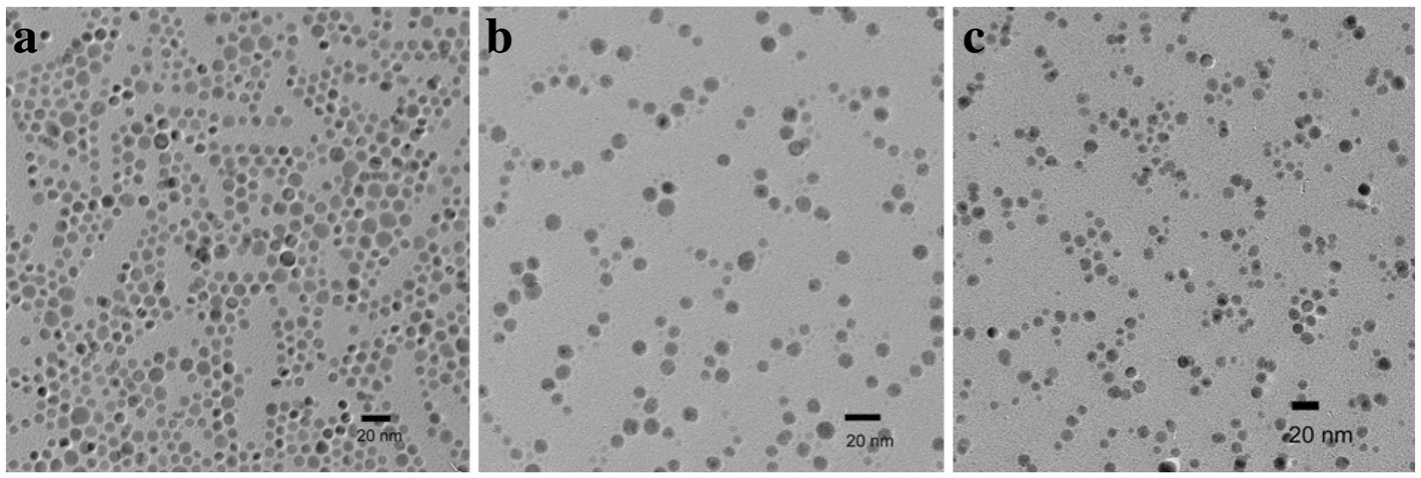
**TEM images of surface-coated silver nanoparticles prepared at 100 °C.** (**a**) AgDDP12, (**b**) AgDDP16, and (**c**) AgDDP18.

Figure 3 shows the TEM images of silver nanoparticles prepared at 100°C and coated also with DDP12, DDP16, and DDP18. The influence of the chain length on the particle size of the silver nanoparticles is not significant compared with that at 60°C. These spherical silver nanoparticles prepared at 100°C display a narrow size distribution (average sizes = 7 to approximately 8 nm) as shown in Figure 3. The silver nanoparticles prepared at 100°C show a smaller size and more uniform scale than those prepared at 60°C. Especially, silver nanoparticles coated with DDP16 or DDP18 synthesized at 100°C appear clearly with the narrow size distribution compared with those prepared at 60°C.

The metal content of the different silver nanoparticles are determined by TG analysis and summarized in Table 1. The metal contents of the silver nanoparticles were found to be correlated with the chain length of coated dithiophosphates and reaction temperature. As the reaction temperature increased, the silver content of the nanoparticles increased. In contrast, the lower the metal content of the silver nanoparticles at the same reaction condition, the shorter the chain length of coated dithiophosphates. The difference of the stability of the coating layer outside is supported by the difference of the decomposition point of DDP12 (222.5°C), DDP16 (260.9°C), and DDP18 (276.5°C) determined by TG analysis.

**Table 1:** Metal contents of the nanoparticles prepared at different temperatures

**Sample**	**Prepared temperature (°C)**	**Mental content (%)**
AgDDP12	60	58.4
AgDDP16	60	45.1
AgDDP18	60	37.7
AgDDP12	100	53.1
AgDDP16	100	48.4
AgDDP18	100	44.4

**Figure 4:**
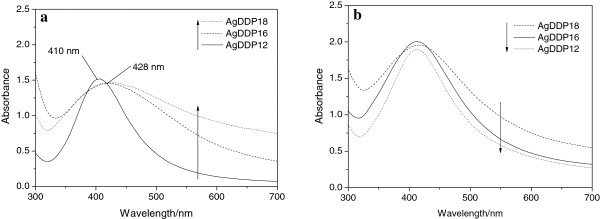
**UV-vis spectra of different silver nanoparticles.** (**a**) Prepared at 60°C and (**b**) 100°C.

The dithiophosphate headgroups coordinate strongly with the silver particles to prevent the nanoparticles from aggregation. In the meanwhile, the outward hydrocarbon tails keep the oily dispersion of the surface-coated silver nanoparticles. The DDP-coated silver nanoparticles exhibit good dispersity in the kerosene and show a typical surface plasmon peak at the wavelength range from 400 to 430 nm. Figure 4 gives the UV–vis spectra of the silver nanoparticles prepared at 60°C and 100°C and then dispersed in kerosene. Because of the relatively wide size distribution of the nanoparticles, the absorption curves of AgDDP16 and AgDDP18 show broader absorptions and red shifts of *λ*_max_ compared with those of AgDDP12, which are consist with the TEM investigations shown in Figures 2 and 3. As shown in Figure 4b, the UV–vis spectra of the silver nanoparticles prepared at 100°C with narrow size distributions give similar peaks without obvious shifts of *λ*_max_.

### Thermal stability of the silver nanofluids

**Figure 5:**
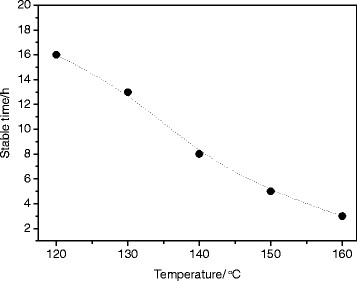
Thermal stability time as a function of temperature for the silver nanofluids.

Thermal stability of the silver nanofluids of AgDDP12 and kerosene was monitored by the UV–vis spectral analysis [18]. The silver nanofluids were kept in an 80-mL teflon-lined stainless steel autoclave which was sealed and thermostated at a given temperature (120°C to approximately 160°C). A small aliquot of the samples was removed from the reactors at fixed time intervals for the spectroscopic analyses. The characteristic absorption for the silver plasmon band in the UV–vis spectra appears at 410 nm. When the absorption values of *λ*_max_ at 410 nm show no visible change, the silver nanofluids are considered to be stable. The suddenly decreased absorption value indicates that the silver nanoparticles in the nanofluids start to aggregate and deposit. The thermal stability time as a function of temperature is shown in Figure 5. It is shown that the silver nanofluid has a stable time of 3 h even at 160°C. The silver nanofluids are stable in a certain time limit at high temperatures. After the time limit, the silver nanoparticles agglomerate and deposit in the fluid quickly at each temperature. The higher the heated temperature, the shorter the thermal stability time.

### Effects of silver nanoparticles on the thermal oxidation stability of kerosene

**Figure 6:**
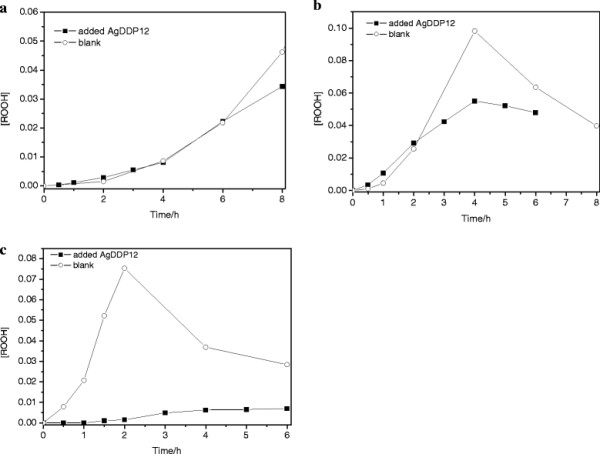
**Comparisons of hydroperoxide concentrations in silver-kerosene nanofluids and blank kerosene thermally oxidized at different temperatures.** (**a**) 120°C, (**b**) 140°C, and (**c**) 150°C.

A stable nanofluid was obtained by dispersing AgDDP12 in kerosene. Effects of the silver nanoparticles on the thermal oxidation stability of kerosene were investigated with the existence of oxygen. Figure 6 gives the hydroperoxide concentration as a function of time in the silver-kerosene nanofluids and the blank kerosene thermally oxidized at 120°C, 140°C, and 150°C. As shown in Figure 6a, there are no obvious differences on hydroperoxide concentration between the silver nanofluid and the blank kerosene oxidized at 120°C, which means that the addition of the silver nanoparticles has little influence on the thermal oxidation of the kerosene. In Figure 6b, it is clear that the hydroperoxide concentrations in the silver nanofluids are lower than those in the blank kerosene during the thermal oxidation process at 140°C. As seen in Figure 6c, there is an apparent decrease of the hydroperoxide concentrations in the silver nanofluid at 150°C. The higher the heated temperature, the more obvious the inhibition effect on the kerosene. When the temperature is high enough, the silver nanoparticles can significantly reduce the formation of the hydroperoxides in the kerosene. It should be ascribed to the coatings opened from the surfaces of the silver nanoparticles, giving the opportunity for oxygen molecules to react with the silver cores directly. The silver nanoparticles react with the oxygen molecules before the kerosene is oxidized and form silver oxide [19]. It indicates that appropriate amounts of silver nanoparticles added in kerosene can enhance its thermal oxidation stability.

## Conclusion

For the reduction synthesis of silver nanoparticles, we use silver nitrate, ascorbic acid, cyclohexylamine, and dialkyl dithiophosphates of various chain lengths (DDP12, DDP16, DDP18), respectively, to serve as precursor, reducing agent, additive, and surface coating reagent. The effects of the chain length of dialkyl dithiophosphate on size and dispersibility of the obtained silver nanoparticles are different. The silver nanoparticles coated with DDP show typical hydrophobicity. DDP12 is better than DDP16 and DDP18 for coating and forming small and uniform silver nanoparticles. The silver nanofluids are stable in a certain time limit at high temperatures because of the surface coating of the ligands. The silver nanoparticles surface-coated by dithiophosphates with good oil dispersity are thus suitable for preparing oil-based nanofluids, which is in favor of the thermal oxidation stability of oil at higher temperatures.

## Competing interests

The authors declare that they have no competing interests.

## Authors’ contributions

DL conceived of the study, carried out the experimental analyses, performed the XRD, UV–vis and TG analyses, TEM characterizations, and drafted the manuscript, WF conceived the study and participated in its design and coordination. Both authors read and approved the final manuscript.
